# Biotechnological Production of Docosahexaenoic Acid Using *Aurantiochytrium limacinum*: Carbon Sources Comparison And Growth Characterization

**DOI:** 10.3390/md13127064

**Published:** 2015-12-05

**Authors:** Sergi Abad, Xavier Turon

**Affiliations:** Bioengineering Department, IQS, Ramon Llull University, Via Augusta 390, Barcelona 08017, Spain; sergi.abad@iqs.edu

**Keywords:** glycerol, biodiesel, DHA, fatty acids, PUFA, omega-3 fatty acids, microalgae, bioprocess, nutraceutics

## Abstract

*Aurantiochytrium limacinum*, a marine heterotrophic protist/microalga has shown interesting yields of docosahexaenoic acid (DHA) when cultured with different carbon sources: glucose, pure and crude glycerol. A complete study in a lab-scale fermenter allowed for the characterization and comparison of the growth kinetic parameters corresponding to each carbon source. Artificial Marine Medium (AMM) with glucose, pure and crude glycerol offered similar biomass yields. The net growth rates (0.10–0.12 h^−1^), biomass (0.7–0.8 g cells/g Substrate) and product (0.14–0.15 g DHA/g cells) yields, as well as DHA productivity were similar using the three carbon sources. Viable potential applications to valorize crude glycerol are envisioned to avoid an environmental problem due to the excess of byproduct.

## 1. Introduction

The potential use of crude glycerol to cultivate oleaginous microorganisms has grown in popularity, essentially to reduce cultivation costs. Simultaneously, the need to valorize glycerol as a co-product of biodiesel has arisen as a result of the biofuel boom. Depending on the feedstock and the process used to produce biodiesel, the contaminants present in crude glycerol vary. The most common ones are methanol and soap [1,2], but also high salinity, which precludes many potential uses of crude glycerol. Salinity is typically a consequence of the catalyst used in the process. Traditionally, the glycerol has been purified to a technical- or pharma-grade (pure) glycerol depending on the degree of contaminants removed. Purified glycerol has been primarily used in the pharmaceutical, food and cosmetic industries. However, the purification costs on a large scale have made its use economically unviable [3,4]. As a consequence, tons of raw glycerol need to be valorized or classified as industrial waste.

### 1.1. Crude Glycerol Valorisation

Previous efforts to use crude glycerol in bioprocesses include cultures of oleaginous microorganisms that accumulate intracellular lipids. Such lipids are then extracted and transesterified to biodiesel [5,6,7]. However, the economic viability of such a closed loop carbon cycle is doubtful. Other bioprocesses based on crude glycerol as the carbon source and using aerobic/anaerobic cultures are being developed. The application, which initially attracted more attention, used single cells as biocatalysts to obtain monomers and building blocks. The best examples are 1,3-propanediol (PDO) [8,9,10,11,12,13] or polyhydroxyalkanoates [14,15,16,17,18]. Other efforts attempted to obtain higher quantities of value-added metabolites, such as pigments [16,19] and long chain polyunsaturated fatty acids (PUFAs), for example arachidonic acid (AA), eicosapentaenoic acid (EPA) and DHA [20,21,22,23,24]. PUFAs are the bioproduct with the highest value.

### 1.2. Crude Glycerol to Obtain Long Chain Poly Unsaturated Fatty Acids

The high salinity of crude glycerol has undesired effects on many organisms, as well as in process units. However, it favors the use of marine microorganisms. Our ongoing research focuses on the production of LC-PUFAs using heterotrophic marine microalgae/protists. Such molecules, considered nutraceutics, have high value due to their health benefits for humans [20]. At the same time, the traditional source of PUFAs, fish oil, is considered a limited resource with a substantial variability in composition and quality. Fish oils are also susceptible to contamination with lipophilic organic chemicals that are now ubiquitous contaminants of marine ecosystems [25,26,27,28]. Algae-oil offers much higher quality [28,29] and commercial applications have been launched.

A family of marine heterotrophic microalgae was selected: thraustochytrids. Many strains of thraustochytrids accumulate considerable amounts of triacylglycerols with a high proportion of long chain polyunsaturated fatty acids (PUFAs), particularly DHA and docosapentaenoic acid (DPA). Its high ratio of DHA, combined with lower amounts of structurally related PUFAs (compared to other species) simplifies the separation and purification of DHA [30]. *Aurantiochytrium limacinum* (*A. limacinum*) SR21 (formerly *Schizochytrium limacinum* SR21) [31] was selected as the main strain used in the current study. Several publications report the capability of thraustochytrids, and specifically *A. limacinum* to synthesize DHA. Many of these focus on the isolation, identification and the ability of the strain to produce DHA [30,32,33]. These studies are based on flask culture assays, which give strain fundamental information, but offer few insights into the bioprocess development potential. Other studies using flask cultures and lab-scale fermenters have proved that *A. limacinum* can effectively grow and produce DHA using pure glycerol as the carbon source [33,34,35,36]. Few attempts went further, assaying with crude glycerol [22,23,37]. When using crude glycerol, the effect of contaminants (e.g., methanol and soap) must be taken into account, as they typically cause a decrease in the final DHA productivity.

At this point, crude glycerol as a valid source for medium–large scale production has to be benchmarked. On the one hand, a comparison with two common carbon sources, glucose and glycerol, is needed using the same strain and culture conditions. Given the difference in purchasing price, their suitability has to be tested. On the other hand, a full kinetic characterization of *A. limacinum* in a fermenter or bioreactor is required. This has never been reported in the literature and is essential for successful development of batch, fed-batch or continuous processes.

## 2. Results and Discussion

With the aim of exploring the industrial potential of thraustochytrids strains as heterotrophic microalgae for the enhanced production of PUFA (mainly DHA), the project was initiated to evaluate the performance of these microorganisms in bioreactors using simple carbon sources. To our best knowledge, a comparison using the three main carbon sources in parallel to report their growth kinetic parameters in fermenters has not been published.

**Figure 1:**
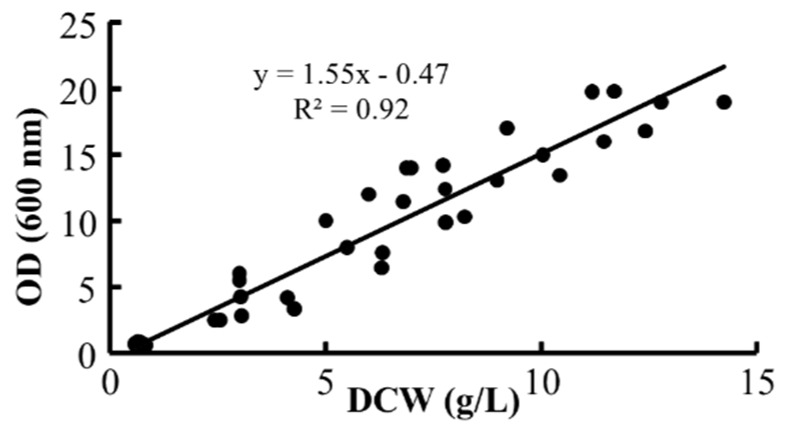
Dry cell weight *vs.* OD curve. The linear relationship was used to determine the dry cell weight of the fermentations performed in the present work.

The first step was to determine the relationship between biomass dry cell weight (DCW) and OD (600 nm). Figure 1 illustrates this relationship, and allows the interpolation of absorbance values to dry weight using pure glycerol. The linear relationship was *y* = 1.55*x* − 0.47, with a positive correlation of 0.92. Cultivations using glucose and crude glycerol showed identical values for the absorbance-dry weight curve. A full characterization in terms of typical growth kinetics and DHA production was then performed using each carbon source.

### 2.1. Growth Rate

**Figure 2:**
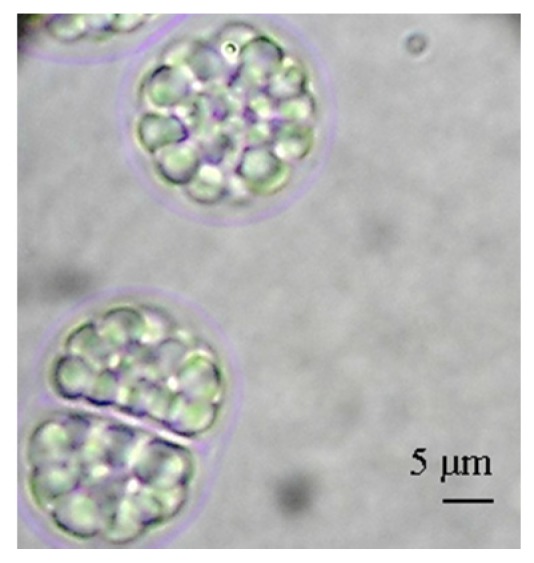
Image of *A. limacinum* sporangium. Sporangium is full of zoospores ready to be released.

The complex life cycle of *A. limacinum* can be simplified by dividing its growth into the zoosporic phase and the vegetative phase. The strain used in the present work can release up to 32 zoospores per cell. Figure 2 shows a sporangium cell that is about to release zoospores. In an effort to describe the growth with a classical model (*i.e.*, Monod), the growth kinetic parameters were determined.

**Figure 3:**
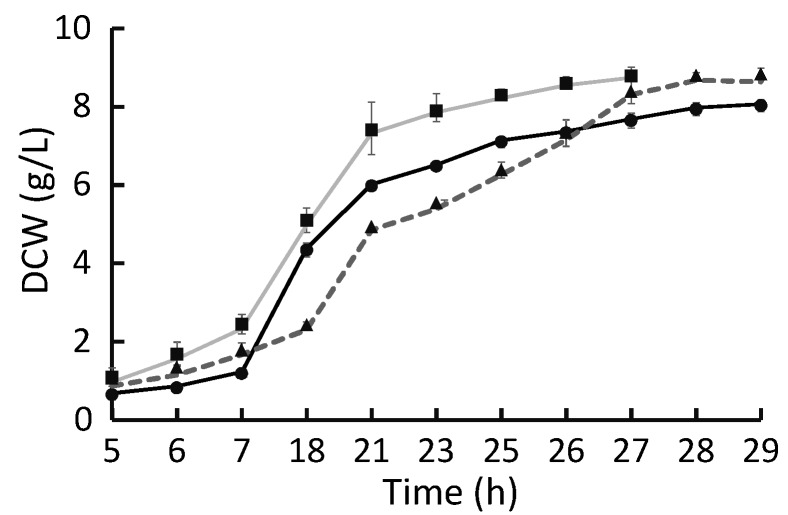
Cultivation of *A. limacinum* using 10 g/L of carbon source: glucose (▲ dotted line), pure glycerol (• black line) and crude glycerol (▪ grey line). Error bars indicate the variability of the different repetitions.

As *A. limacinum* released zoospores, a peak in cell formation was detected, leading to the highest growth rate (μ_max_) values in the batch. When glucose was used, higher maximum growth rates were achieved (0.21 ± 0.02 h^−1^) compared to glycerol (0.18 ± 0.01 h^−1^) and crude glycerol (0.19 ± 0.02 h^−1^). On the other hand, when *A. limacinum* reached the vegetative phase, the growth mechanism was restricted to bipartitions. Therefore, the growth rate declined. Net growth rates were slightly higher in glycerol cultures (0.12 ± 0.01 h^−1^) compared to glucose (0.10 ± 0.02 h^−1^) and crude glycerol (0.10 ± 0.01 h^−1^). In conclusion, more constant growth rates were found when glycerol/crude glycerol was used, as seen in Figure 3. Data in Figure 3 correspond to the average value of triplicates for glucose, and quadruplicates for glycerol and crude glycerol. Standard deviations were determined using the values of different repetitions. The presence of impurities from crude glycerol (at the concentration used in these cultures) did not inhibit growth.

### 2.2. Growth Yield

Growth yield is a crucial parameter when different carbon sources are evaluated. This comparative study revealed similar yields using the three carbon sources, as summarized in Table 1. Crude glycerol rendered the highest growth yield. Crude glycerol contains several impurities from the transesterification (unreacted feedstock, catalyst, and other byproducts). Typically, 75% to 85% of its weight corresponds to glycerol (83% in the sample in our study). Unreacted feedstock (used cooking oils) might add some nutrients, leading to an increased yield.

**Table 1:** Summary of the growth kinetics parameters found using different carbon sources.

	Glucose		Glycerol		Crude Glycerol	
*X* (g/L)	8.83	±0.15	8.21	±0.17	8.86	±0.12
*Y*_X/S_ (g/g)	0.84	±0.05	0.74	±0.04	0.85	±0.1
μ_max_ (h^−1^)	0.21	±0.02	0.18	±0.01	0.19	±0.02
μ_net_ (h^−1^)	0.10	±0.005	0.12	±0.01	0.10	±0.01
K_S_ (g/L)	2.5	±0.5	5.0	±1.5	8.0	±1.5
*Y*_P/X_ (g/g)	0.14	±0.02	0.15	±0.04	0.15	±0.02

In the literature, the disparity of values reported was found to be really high, ranging from values as low as 0.2–0.3 g dcw/g carbon source [22,23,24,30] to high-end values of 0.6–0.8 g dcw/g carbon source [31,34,38]. Values corresponding to *A. limacinum SR21* obtained at bioreactor scale (not flask) are summarized in Table 2. Another similar study, but using a different strain (*A. limacinum mh0186*) and glucose, reported a growth yield of 0.53 g dcw/g glucose, 0.11 h^−1^ of specific growth rate and 0.22 g DHA/g dcw as DHA yield. The results reported in this contribution are valuable because they provide a comparison performed in identical conditions. Therefore, they contribute to the general understanding of the process at a reactor scale.

**Table 2:** Comparison of the growth kinetics parameters using: *A.limacinum SR21* and any of the three carbon sources, in batch lab-scale bioreactor.

Carbon Source	Time (h)	So (g/L)	*X* (g/L)	*Y*_x/s_ (g/g)	*Y*_p/x_ (g/g)	μ_net_ (h^−1^)	Reference
Glucose	125	150	59.2	0.39 *	0.26 *	0.05 *	[39]
	50	40	21	0.52 *	0.22 *	0.11 *	[35]
	100	120	50	0.41 *	0.29 *	0.11	[40]
	30	10	8.83	0.84	0.14	0.10	This study
Glycerol	198	100	32.8	0.33 *	0.18 *	0.023 *	[37]
	30	10	8.21	0.74	0.15	0.12	This study
Crude glycerol	30	10	8.86	0,85	0.15	0.10	This study

* denotes a calculated parameter from the reported data in the reference.

Only one study that used a bioreactor, the same strain and crude glycerol was found in the literature, with a growth yield of 0.26 g dcw/g glucose, 0.69 day^−1^ (0.028 h^−1^) as maximum specific growth rate and 0.14 g DHA/g dcw as DHA yield [22]. The results of growth are notably lower than the current study, although the DHA yield is comparable.

Nitrogen source has an important role in growth and a lack of nitrogen can reduce the carbon source yield. Organic nitrogen sources are preferred in thraustochytrids cultivations [22,41]. Yeast extract and tryptone are typically used in the bibliographic references, and were the choice as well for this study.

### 2.3. Affinity Constant

The affinity constant is the hardest growth parameter to characterize in batch reactors, and typically presents more inaccuracies. In this study, the cultivation of an eukaryotic species with a complex life cycle (compared to other microbial strains) added more difficulties. As seen in Table 1, *A. limacinum* has higher affinity to glucose (2.5 ± 0.5 g S/L) compared to pure (5 ± 1.5 g S/L) and crude (8 ± 1.5 g S/L) glycerol. *A. limacinum* shows a lower affinity for crude glycerol compared to pure glycerol and glucose.

Growth rates and yields are not affected by different values of the affinity constant. However, K_S_ becomes an important parameter when designing continuous cultures. However, the different cell cycles taking place in batch and in steady state of a continuous reactor has to be considered. Ks values determined in a steady state of a chemostat could undergo significant variations, where mainly vegetative cells are found. Taking these values into consideration, we can affirm that *A. limacinum* has a metabolic preference towards glucose. Even so, glycerol and, surprisingly, crude glycerol are perfectly valid alternatives to develop a bioprocess. The availability and especially the cost of the nutrients may determine the choice when lipids and DHA have to be produced at a large scale.

### 2.4. DHA Yield

**Figure 4:**
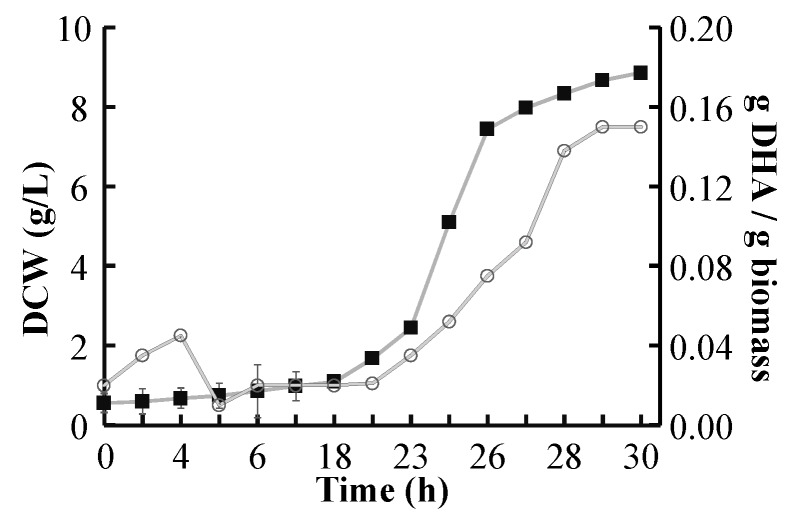
Evolution of cells and DHA production in batch using crude glycerol. Full squares indicate biomass concentration and circles DHA yield. Error bars indicate the variability of the different repetitions.

The product yield is crucial when pursuing high productivities. In this study, glycerol-based fermentations rendered slightly higher DHA yield (Y_P/X_). Crude and pure glycerol yielded around 0.15 g DHA/g cells. Meanwhile, glucose cultures yielded an average of 0.14 g/g. DHA content was monitored during the cultivation, data included in Figure 4 from triplicate cultures (biomass curve) and duplicated DHA analysis for yield determination. Standard deviation was determined using the values of different repetitions. The intracellular accumulation of lipids and DHA is clearly related to cell growth state. Figure 4 shows an initial low yield of DHA (below 5% of cell weight), which decreased as cells started the exponential growth phase. Triglycerides (TG) are essentially an energy sink, among other physiological and functional usages. Cells starting their exponential growth use the internal energy stored in their TG to generate zoospores, decreasing the overall DHA yield. Then, when the microorganism switches to the vegetative state, it tends to accumulate lipids again, including DHA. This observation is aligned with the literature, where the formation of zoospores was reported to consume high amounts of energy [31,42].

With the nutrient concentrations and working parameters used in this study, a final concentration of 1.24 g DHA/L was reached using glucose, 1.23 g DHA/L for pure glycerol, and 1.33 g DHA/L using crude glycerol. The results indicate that DHA production is not affected by the carbon source used.

The current state of the art shows a notable disparity in terms of DHA productivity, especially in glycerol cultures. When glucose was used, the yields reported varied from 0.23 to 0.26 g DHA/g glucose [36,39,40]. When pure or crude glycerol was used, the variability was much larger. The strain used, but mainly the glycerol source and process parameters lead to considerable variations in the yield. Precisely, improved or optimized growth media composition, together with fatty acid stimulation via oxygen deprivation and/or variations in the carbon to nitrogen ratio (*C*/*N*), also increase the final DHA yield. Jakobsen *et al.* obtained a high productivity of 90 mg DHA/l-h with pure glycerol and stressing cells using nitrogen starvation [34]. Studies using glucose in batch reactor reported productivities ranging from 116 to 129 mg DHA L^−1^·h^−1^ [36,39,40]. Reports of productivities using pure glycerol range from 38 to 90 mg DHA L^−1^·h^−1^ [30,34]. Crude glycerol references reported DHA productivities from 21 to 23 mg DHA L^−1^·h^−1^ [22,23,43]. Glucose-based fermentations reporting superior productivities rely on higher initial substrate concentration, thus reaching final higher cell concentrations. Crude glycerol fermentations typically must use less concentrated media, in order to avoid accumulation of inhibiting toxic compounds. This leads to lower final cell concentration and lower production. The productivities reached in the present study are similar with all carbon sources. Values obtained using glucose and pure glycerol were 55 mg DHA L^−1^·h^−1^, in the range of the reported productivities for glycerol, but lower in the case of glucose. As mentioned, such lower productivities for glucose are explained due to the lower initial concentrations compared to the literature values. Productivities for crude glycerol average 60 mg DHA L^−1^·h^−1^, which significantly improves the reported bibliographic values. An optimization of the growth media and operational parameters will contribute to further increase the productivity of the process with any of the carbon sources.

## 3. Experimental Section

### 3.1. Strain and Culture Media

*Aurantiochytrium limacinum* (ATCC MYA-1381) from the American Type Culture Collection was used in this study. The culture media used artificial marine water (AMM), as reported in [22]. One gram per liter of both yeast extract and tryptone were used as nitrogen source. AMM with 15% glycerol was used for long term conservation of the microorganism at −80 °C. A carbon source was added to AMM, to a final concentration of 10 g/L, in order to establish substrate comparison. Glucose (d(+)Glucose Anhydrous (Scharlau, Barcelona, Spain), pure glycerol (Fisher Scientific Madrid Spain, purity 99.5% min.), and crude glycerol (provided by Transportes Ceferino Martínez S.A., Vilafant, Spain) were used. Crude glycerol was obtained after the alkali process using cooking oil (UCO) to produce biodiesel by a transportation company. The glycerol content was 83%.

### 3.2. Cultivation

Inoculums were prepared using 40 mL AMM + carbon source and incubated at 20 °C under shaking conditions (200 rpm). After 48 h, cells were used to inoculate the bioreactor at a 2%–2.5% volume ratio, obtaining *ca.* 0.5 of initial OD. Cultures were grown in a bench scale 2.5 L bioreactor, Minifors, Infors (Bottmingen, Switzerland), operated in batch using 1.5 L as working volume. The fermenter was stirred using a Rushton impeller turbine. An aeration rate of 1.5 vvm was used in all experiments. Samples consisting of 10 mL of the fermentation broth were taken every 2 h. Samples were used to monitor cell growth by evaluating OD_600_ (Jenway 6310 spectrophotometer, Jenway Staffordshire, UK), dry weight of biomass, residual glycerol and glucose concentrations. All assays were carried out in triplicate

### 3.3. Glucose Cultivation

Cultures were grown using AMM and 10 g/L of glucose as carbon source. Triplicate cultures were monitored in terms of pH, temperature, % dissolved oxygen and agitation (RPM) to guarantee identical growth conditions. The growth kinetics parameters measured are summarized in Table 1. At the end of the exponential growth phase, samples were withdrawn and processed: cell lysis, lipid extraction and triglycerides methylation to fatty acid methyl esters (FAMEs). The DHA yield obtained is included in Table 1 as the ratio between the DHA quantified by GC-FID and the dry biomass used.

### 3.4. Glycerol Cultivation

Cultures using glycerol used growth media, as described in Section 3.1, but with 10 g/L of pure glycerol or 10 g/L of total glycerol when using crude glycerol (calculated with the 83% purity as analyzed). Each culture had 4 replicates controlling operating parameters as in glucose cultures. Samples of biomass harvested at the end of the batch were analyzed to quantify DHA.

### 3.5. Analysis

Dry weight of biomass was determined after centrifugation of 10 mL of the sample (Sigma 1–14 Microfuge) at 4000 RPM for 15 min. The pellet was washed with a saline solution (5 g/L) and centrifuged again. Then, it was lyophilized (LyoAlfa 6 freeze drier, Telstar, Barcelona, Spain) and finally weighted.

Residual glycerol was evaluated using a liquid chromatography protocol as detailed in Abad *et al.* [44]. Residual glucose concentration was measured by DNS [45].

DHA content quantification was fully validated, consisting of two consecutive steps: preparation of FAME and chromatographic analysis. FAMEs were synthesized by a four in one step procedure. The method was adapted from Indarti *et al.* (2005) [46] and conveniently modified to use freeze-dried microorganisms. In a 2 mL glass vial, samples were weighed (5 mg) and dissolved in 500 μL fresh methanol, sulfuric acid and chloroform (1.7:0.3:2 *v/v/v*) mixture. The vials were sealed with aluminum crimp caps to avoid volume loss during the reaction. Samples were maintained in a water bath at 80 °C for 30 min. Then, 100 μL of water were added to separate two phases. FAMEs are found dissolved in chloroform.

Afterwards, chloroform phase was collected and analyzed by HRGC 7890GC (Agilent technologies, Germany) equipped with a flame ionization detector and Supelco SP™-2380 (60 m × 0.25 mm × 0.20 μm) column. Helium was used as carrier gas. The temperature settings for injector and detector were 250 °C. The fatty acids were identified by comparing the retention times with those of standard fatty acids (Sigma-Aldrich, Madrid, Spain).

The overall DHA quantification method (sample processing and HRGC-FID) analysis was successfully validated as the linearity, accuracy and repeatability were determined using a calibration curve of eight different DHA standard concentrations (Sigma-Aldrich, Saint Louis, MI, USA) in triplicates coupled with tricosanoic acid (C23:0) as internal standard. The results stayed within a noteworthy coefficient of variation (CV) of 5% (data included in Supplementary Materials).

### 3.6. Kinetic Parameters Calculation

Specific growth rate (μ, h^−1^) was evaluated on the basis of values of biomass at the exponential phase of growth as expressed in Equation (1).

(1) $$\frac{dX}{dt}=\mu_{g}\cdot X$$

The maximum specific growth rate μ_max_ (h^−1^) was the maximum of the specific growth rates calculated during the exponential growth phase, as expressed in Equation (1).

The net specific growth rate μ_net_ (h^−1^) was calculated between the inoculation and the end of the exponential growth phase using Equation (1). Therefore, μ_net_ includes the lag phase.

The growth yield (*Y*_X/S_) was calculated as the amount of biomass obtained compared to the substrate consumed, as in Equation (2).

(2) $$Y_{X/S}=\frac{dX}{dS}$$

where *X* stands for biomass concentration (g/L) and *S* for substrate concentration (carbon source, g/L).

In this case, the substrate was the main carbon source used (glucose or glycerol). Contributions in terms of carbon from organic nitrogen sources or other nutrients (*i.e.*, tryptone, yeast extract and crude glycerol) could be metabolically used as well by cells; however, only the main carbon source added was used in all calculations. This might be particularly important when using crude glycerol as carbon source because it typically contains impurities.

The specific DHA yield (*Y*_P/S_), which is the amount of DHA obtained (GC-FID analysis) per gram of biomass, was calculated as in Equation (3).

(3) $$Y_{P/X}=\frac{dP}{dX}$$

where *P* stands for product mass (g) and *X* for biomass (g).

The affinity or semi-saturation constant or K_S_, is typically defined as the concentration of substrate leading to a μ_*g*_ = *1/2* μ_*max*_. The value reported was the average of all the calculated K_S_ corresponding to the different repetitions. K_S_ was determined by graphical approach derived from the Monod equation in batch culture according to Equation (4),

(4) $$\frac{1}{\mu_{g}}=\frac{K_{S}}{\mu_{m}}\cdot \frac{1}{S}+\frac{1}{\mu_{m}}$$

where μ_g_, the specific growth rate, was calculated using Equation (1); S is the substrate concentration and μ_max_ is the maximum growth rate. A double reciprocal plot (1/μ_g_ *vs.* 1/*S*) was built, where the calculated slope corresponds to $\frac{K_{S}}{\mu_{m}}$.

All the calculated parameters are summarized in Table 1.

## 4. Conclusions

*A. limacinum* was successfully cultured in a bioreactor operated in batch to fully characterize growth kinetics parameters using glucose as well as pure and crude glycerol. *A. limacinum* was confirmed as a suitable microorganism for effectively producing DHA using any of the three carbon sources. *A. limacinum* has become a potential industrial microorganism for producing lipids and PUFAs. The results demonstrated that an industrial byproduct, like crude glycerol, could be used as carbon source in such a bioprocess. *A. limacinum* showed a robust behavior, with a similar performance when glucose as well as pure or crude glycerol was used. Algae-oil also leads to contaminant-free production of DHA compared to fish oil (mainly due to biomagnification of oceanic persistent organic pollutants (POPs)). Nevertheless, deeper analysis is needed to guarantee the absence or minimal concentrations of POPs.

## Supplementary Files

Supplementary File 1
