# Supplementary material for: Biotechnological Production of Docosahexaenoic Acid Using Aurantiochytrium limacinum: Carbon Sources Comparison And Growth Characterization
Source: Mar Drugs. 2015 Dec 5;13(12):7275–84. doi: 10.3390/md13127064 (PMC4699237; doi:10.3390/md13127064)
Supplement: Supplementary File 1 [file marinedrugs-13-07064-s001.docx]

**Supplementary Materials: Biotechnological Production of Docosahexaenoic Acid Using *Aurantiochytrium limacinum*: Carbon Sources Comparison and Growth Characterization**

Sergi Abad and Xavier Turon

DHA Determination and Quantification Methodology (Sample Preparation and
HRGC Analysis)

The method is a modification of Indarti *et al.* adapted to extract and transform triglycerides to methylated fatty acids (FAMEs) from microorganisms. In a 2 mL glass vial, lyophilized biomass was weighed (5 mg) and dissolved in 500 µL fresh methanol, sulfuric acid and chloroform (1.7:0.3:2 *v/v/v*) mixture. The vials were sealed with aluminum crimp caps to avoid volume los during the Fischer reaction. To do so, samples were maintained in a water bath at 80 °C during 30 min. After that samples were cooled during 2 min at room temperature. Then 100 µL of water were added to separate two phases, having FAMEs dissolved in chloroform.

Chloroform phase was collected and analyzed by HRGC 7890GC (Agilent technologies, Waldbronn, Germany) equipped with a flame ionization detector and Supelco SP™-2380
(60 m × 0.25 mm × 0.20 μm) column. Helium was used as carrier gas. The temperature settings for injector and detector were 250 °C. The fatty acids were identified by comparing the retention times with those of standard fatty acids (Sigma-Aldrich, Madrid Spain).

The method was first evaluated to find the relative maximum percentage extraction, using the same lyophilized biomass sample. After exploring different conditions using two Design of Experiments (DoE) based on Taguchi’s orthogonal matrices, the optimum conditions were set at 85 °C and 35 min.

Once the method was established it was validated in two steps. First, the capabilities of the sample preparation procedure were validated using three standards: methyl palmitate (MP), palmitic acid (P) and glyceril tripalmitate (PPP) (Sigma-Aldrich, Madrid Spain). The procedure offers nearly a 100% of recovery in any case as shown in Table S1.

**Table S1.** Calibration curves of MP, P and PPP.

| **[MP]** | **Area** | **CV (%)** | **Fr** | **[P]** | **Area** | **CV (%)** | **Fr** | **[PPP]** | **Area** | **CV (%)** | **Fr** |
| --- | --- | --- | --- | --- | --- | --- | --- | --- | --- | --- | --- |
| 2.84 | 2043 | 0.2 | 719.4 | 2.5 | 1752 | 4.5 | 701 | 2.5 | 1754 | 0.8 | 702 |
| 5.2 | 3599 | 0.7 | 692.1 | 5 | 3661 | 2.8 | 732 | 5 | 3695 | 0.9 | 739 |
| 7.28 | 5149 | 0.2 | 707.2 | 7.5 | 5260 | 3.2 | 701 | 7.5 | 5312 | 0.2 | 708 |
| 10.2 | 7541 | 0.5 | 739.3 | 10 | 7702 | 0.1 | 770 | 10 | 7322 | 1.3 | 732 |
| 12.6 | 8954 | 0.2 | 710.6 | 12.5 | 8995 | 0.9 | 720 | 12.5 | 8745 | 1.3 | 700 |
|  | Fr stats | Mean | 713.7 |  | Fr stats | Mean | 711 |  | Fr stats | Mean | 716.1 |
|  |  | SD | 17.4 |  |  | SD | 28.6 |  |  | SD | 18.2 |
|  |  | CVf (%) | 2.4 |  |  | CVf (%) | 4.0 |  |  | CVf (%) | 2.5 |
|  |  |  |  |  |  | R (%) | 99.7 |  |  | R (%) | 100.3 |
|  |  |  |  |  |  |  |  |  |  | R′ (%) | 100.7 |

In Table 1 the value of *Area* corresponds to the mean of three area measures with the corresponding CV (%) showing the reliability of the measurements. At the right, the response factor (Fr) is defined as the ratio between the concentration of a compound being analyzed and the response of the detector to that compound. Fr stats are listed below its column. The indicated mean value is the average of all the values with the corresponding standard deviation (SD). CVf (%) is the Fr variation coefficient. For P and PPP, R (%) indicates the recovery relative to the MP standard. On the other hand R’ (%) indicates the recovery of PPP relative to P.

As demonstrated, the method can handle the different TG in the sample. Then, the method was evaluated in terms of DHA. As this FA is known to be labile the same standard was evaluated at different times of reaction. As summarized in Table S2, close to 100% of DHA was recovered after the whole sample preparation process, using the reaction conditions established with Taguchi’s orthogonal matrices.

**Table S2.** Results and stats of the sample preparation process applied on a DHA standard with different times of reaction. Experiments were performed at 85 ºC. SD stands for standard deviation; CV stands for Variation coefficient; Fr stands for response factor. R_30_ corresponds to the recovery % relative to 30 min experiment. Mean response equals mean area.

| **Time (min)** | **[DHA] (g/L)** | **Mean** | **SD** | **CV (%)** | **Fr** | **R30 (%)** |
| --- | --- | --- | --- | --- | --- | --- |
| 5 | 3 | 2952 | 43.8 | 1.49 | 984 | 96.3 |
| 10 | 3 | 3064 | 22.6 | 0.74 | 1021 | 98.3 |
| 15 | 3 | 3116 | 4.9 | 0.16 | 1039 | 99.0 |
| 20 | 3 | 3146 | 7.1 | 0.22 | 1049 | 99.4 |
| 25 | 3 | 3165 | 2.1 | 0.07 | 1055 | 99.9 |
| 30 | 3 | 3168 | 0.7 | 0.02 | 1056 | 100.0 |
| 35 | 3 | 3136 | 8.0 | 0.00 | 1045 | 99.0 |

Finally, a set of sequential samples were injected to evaluate statistical attributes of the method investigated. The results are shown in the Table S3.

**Table S3.** Linearity, repeatability, accuracy and selectivity stats from the DHA quantification method.

|  |  | **Precision (Repeatability)** | | **Accuracy** | |  |  |
| --- | --- | --- | --- | --- | --- | --- | --- |
| **[DHA] (mg/mL)** | **Mean Response** | **SD** | **CV (%)** | **Concentration Found (g/L)** | **Recovery (%)** | **DHA** | |
| 0.7 | 701 | 14 | 2 | 0.68 | 96.8 | Sensitivity | No Interference |
| 1.4 | 1522 | 20 | 1.3 | 1.37 | 98.0 | Lineatiy | 0.7–6.1 g/L |
| 2.8 | 3129 | 8 | 0.3 | 2.73 | 97.6 | R^2^ | 0.99 |
| 4.4 | 5070 | 80 | 1.6 | 4.38 | 99.5 | CV (%) | 1.2 |
| 6.1 | 7167 | 59 | 0.8 | 6.15 | 100.9 | LOD/LOQ | 0.15/0.5 (g/L) |
|  |  | Mean (%) | 1.2 | Mean (%) | 98.6 |  |  |
|  |  |  |  | CV (%) | 1.7 |  |  |

Observing the FA profile from *A. limacinum* (obtained using the current method) illustrated in the Figure 1, P is satisfactorily separated from other FA species (Every FA tagged in the Figure S1 was identified by comparing the retention time with a standard mixture of 37 FAME, purchased from Sigma-Aldrich, Madrid Spain) extracted from this microorganism, thus showing an excellent specificity and selectivity. The internal standard used during the validation as well as regular
analysis (to monitor suitability of the whole process for every run) was tricosanoic acid (C23:0)
(Sigma-Aldrich, Madrid Spain) which is not present in natural samples. The internal standard is always added before methylation.


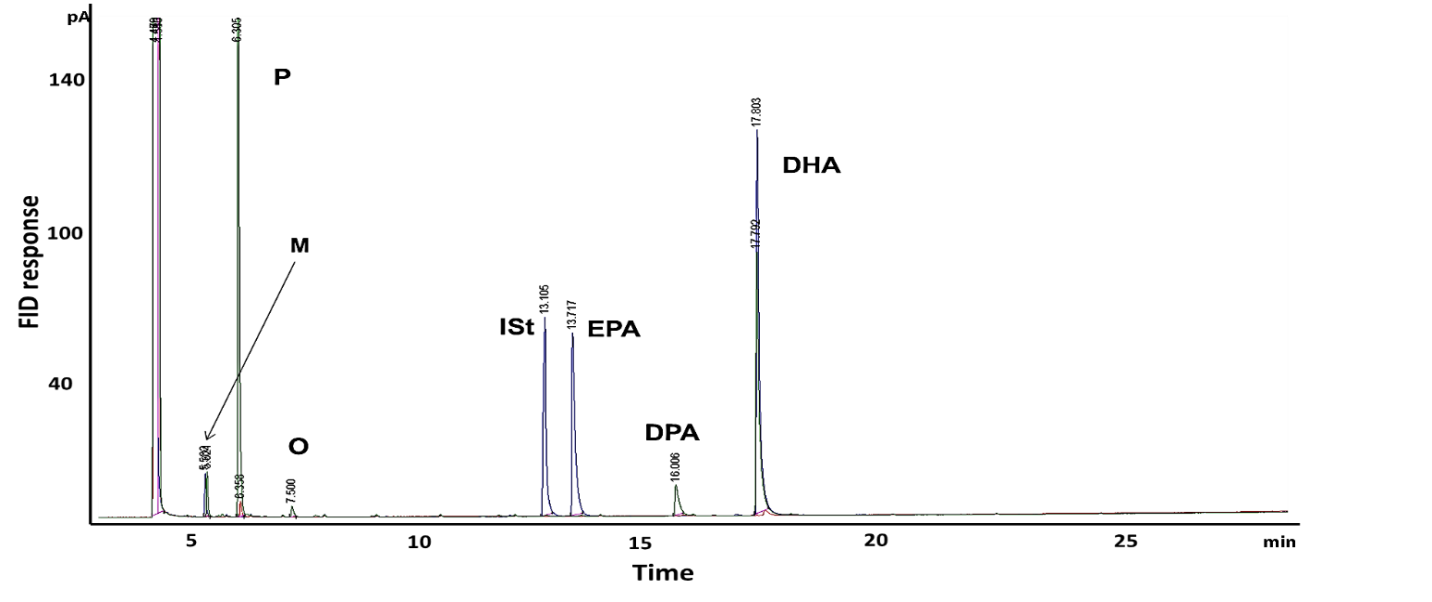


**Figure S1.** Fatty acid profile extracted from *A. limacinum* using the current described method. P is palmitic acid; M myristic acid; O oleic acid. ISt is the internal standard, tricosanoic acid (C23:0).
EPA was added as standard and corresponds to eicosapentaenoic acid. DPA is docosapentaenoic acid and DHA is docosahexaenoic acid. Every peak was identified by injecting Sigma 37 FAME standard.
